# Antigen transfer and its effect on vaccine-induced immune amplification and tolerance

**DOI:** 10.7150/thno.75904

**Published:** 2022-08-01

**Authors:** Yingying Shi, Yichao Lu, Jian You

**Affiliations:** College of Pharmaceutical Sciences, Zhejiang University, 866 Yuhangtang Road, Hangzhou 310058, Zhejiang, China.

**Keywords:** antigen transfer, DCs-based receptor, vaccine, immune amplification, immune tolerance

## Abstract

Antigen transfer refers to the process of intercellular information exchange, where antigenic components including nucleic acids, antigen proteins/peptides and peptide-major histocompatibility complexes (p-MHCs) are transmitted from donor cells to recipient cells at the thymus, secondary lymphoid organs (SLOs), intestine, allergic sites, allografts, pathological lesions and vaccine injection sites via trogocytosis, gap junctions, tunnel nanotubes (TNTs), or extracellular vesicles (EVs). In the context of vaccine inoculation, antigen transfer is manipulated by the vaccine type and administration route, which consequently influences, even alters the immunological outcome, i.e., immune amplification and tolerance. Mainly focused on dendritic cells (DCs)-based antigen receptors, this review systematically introduces the biological process, molecular basis and clinical manifestation of antigen transfer.

## Introduction

Antigen transfer is an important approach of cell-to-cell communication, where antigenic information is actively transmitted from donor cell to recipient cell in the form of nucleic acid, antigen (Ag) protein/peptide, peptide major histocompatibility complex (p-MHC) and vaccine particle mainly at the thymus, peripheral lymphoid organ, intestine, allergic site, allograft, pathological lesion and vaccine injection site through the contact-dependent pathways including trogocytosis, gap junctions, and tunnel nanotubes (TNTs), and the contact-independent extracellular vesicles (EVs) 1. In fact, both professional antigen-presenting cells (APCs) and somatic cells (i.e., non-APCs) are potential participants in antigen transfer. Specifically, Ag can be transferred from APCs to APCs, from non-APCs to APCs, from APCs to non-APCs, and even from non-APCs to non-APCs, which is of vital significance for coordinating immune elicitation/amplification and tolerance establishment/maintenance 2.

Encompassing dendritic cells (DCs), B cells and macrophages, APCs are a heterogeneous family with functionally specialized subsets that mediate innate and adaptive immunity upon local microenvironmental cues. Notably, DCs are the most powerful APCs that accommodate a dual regulatory effect in immune activation and tolerance induction. It has been widely recognized that DCs modulate the activation of T cells through both canonical 3 and non-canonical 4-7 Ag presentation pathways, in which the MHC system is flexibly mobilized to elicit potent immune responses against virus infection 8 and tumorigenesis 9, 10. On the other hand, DCs are paramount in the orchestration of both central and peripheral tolerance. DCs promote central tolerance during the negative selection of autoreactive T cells in the thymus, and induce a tolerogenic or exhausted state of T cells by driving the polarization of regulatory T cells (Tregs) from naïve T cells in the periphery 11. Such versatile immune competence of DCs is largely attributed to their inherent characteristics, such as: **1)** multiple subsets with functionalized phenotypes that constitute a wide-ranging immune surveillance 12, 13, including conventional DC (cDCs) 14-17, Langerhans cells (LCs) 16, 17, plasmacytoid DCs (pDCs) 18, and monocyte-derived DCs (mo-DCs) 19; **2)** rapid sensing and chemotaxis toward sites under the “non-self” invasion 20; **3)** diverse endocytic receptor repertoires and Ag process systems for multi-dimensional activation of T cells 21, 22; and **4)** homing toward the secondary lymphoid organs (SLOs, including lymph nodes (LNs), spleen, Peyer's patches (PPs), adenoids and tonsils) during maturation to provide a timely integration of environmental signals. Moreover, besides direct capture of peripheral Ag 23-26, DCs are capable of collecting antigenic information from Ag-exposed live cells including non-leukocytes and other types or individuals of APCs 14, 23, 27-29, serving as Ag acceptors to ensure an all-round supervision over the body and promote the flexible modulation of immune activation 1 and tolerance 30.

Indeed, the existence of intercellular antigen transfer largely reshapes our understanding about the mode of action of vaccines. For locally administrated vaccines, the accessibility and availability of peripheral Ag by SLOs-resident DCs plays a central role in the in-situ activation of T-/B- lymphocytes and consequently determines the immunological outcomes 31. However, considering the poor mobilization ability of tissue-resident DCs and the potential cell damage caused by the “non-self” attack, a direct contact with the source Ag may be difficult, risky and not necessary. As a matter of fact, APCs and non-APCs predominate at the vaccine sites can both be positively vaccinated and act as intermediaries that provide antigenic information to DCs, such as keratinocytes (KCs) 32, muscle cells 33, LCs 34, 35, migratory DCs 36, macrophages and B cells. As a matter of fact, antigen transfer from infected, transformed, or vaccinated live cells to DCs prevents the risk of cell damage caused by direct virus/tumor contact, compensates the insufficient availability of certain types of DCs to distal Ag, and enhances specific immune responses against natural infection, tumorigenesis and vaccine inoculation 2, 37-39.

Therefore, rationally utilize and regulate antigen transfer for improved vaccine efficacy might demonstrate some clinical significance. However, current understanding about the biological process and molecular basis of antigen transfer is insufficient 40, which may limit the efficiency and safety of current vaccines. Herein, mainly focused on DCs-based Ag receptors, this review systematically introduces the mode, location and participant of antigen transfer, especially in the context of vaccine inoculation, which may provide guidance for the design and development of vaccines.

## Pathological and physiological significance of antigen transfer

### Pathological significance of antigen transfer

Antigen transfer refers to the intercellular trafficking (active behavior) of antigenic information from the donor to the acceptor, which effectively promotes the availability of Ag and extends the breadth and duration of immune response. APCs, as represented by DCs, fail to elicit immune responses when directly exposed to viruses that are highly invasive and cell-destructive (e.g., herpes simplex virus (HSV), Epstein-Barr virus (EBV), cytomegalovirus and some influenza viruses) 37. Likewise, transformed or malignant cells may reshape the microenvironment to inactivate infiltrating immune cells, as both the number and the LNs-migrating ability of tumor-infiltrating DCs drastically decreased with time 41.

Under these circumstances, Ag is transferred from infected or transformed live cells to DCs, which greatly reduces the risk of direct virus/tumor contact and magnifies specific immune response to natural infection and tumorigenesis.

### Physiological significance of antigen transfer

It is reported that compared to APCs, most somatic cells (e.g., muscle cells, keratinocytes) abundant at the sites of vaccine administration can be positively vaccinated and even display higher competence in nucleic acid-transfection and protein-uptake 2. However, these cells are generally low in the expression of co-stimulatory molecules, which deprives their ability of direct T-cell activation upon vaccination. In response to this situation, Ag is transferred from vaccinated somatic cells to the nearby APCs to activate specific immune response.

On the other hand, tissue-resident DCs, important components of the lymphoid organs that far outnumber their circulating counterparts have poor mobilization ability, which greatly limits their accessibility to the peripheral Ag. However, these DCs, LN-resident CD8α^+^ DCs in particular, have been shown to present Ag from other cells (e.g., circulating DCs), leading to efficient elicitation of the cytotoxic T lymphocyte (CTL) response 38, 39. Therefore, antigen transfer from other cells to tissue-resident DCs may compensate the low availability of these certain types of APCs to distal Ag and magnifies specific immune response.

## Mode of antigen transfer

**Figure 1:**
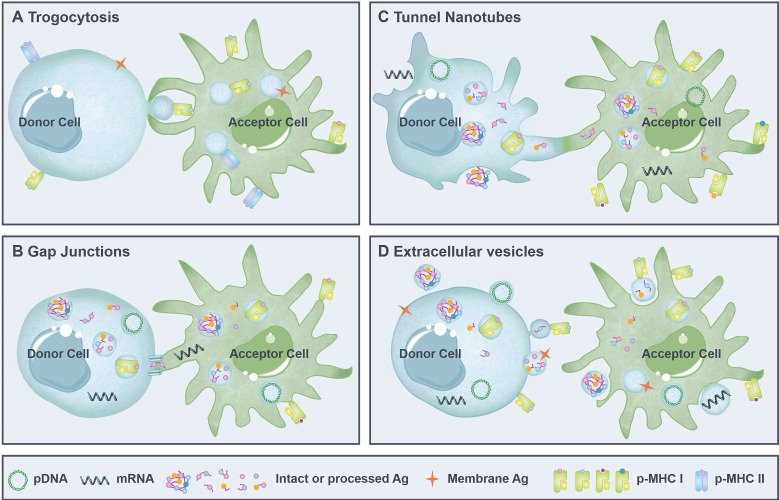
** Four modes of antigen transfer. (A)** Trogocytosis. Cells in close contact can directly "bite" and internalize membrane-associated Ags and/or p-MHCs from each other. **(B)** Gap junction. Adjacent cells exchange intracellular antigenic information (pDNA, mRNA, Ag protein/peptide and p-MHCs) via hexamer channels. **(C)** Tunnel nanotubes (TNTs). Cell-cell connection by actin-based membrane protrusions that establish cytoplasmic continuity between distant cells and enable the exchange of cytoplasmic Ags and cell surface-associated Ags. **(D)** Extracellular vesicles (EVs). Donor cells bud directly from the plasma membrane to generate microvesicles containing p-MHCs and/or membrane-associated Ags, or secrete exosomes derived from the intracellular Ag-incorporating endosomes. These microvesicles and exosomes diffuse into the extracellular space to be captured by acceptor cells. Ag: antigen; mRNA: messenger RNA; pDNA: plasmid DNA; p-MHC I/II: peptide-major histocompatibility complex class I/II molecules.

Intercellular antigen transfer is largely mediated by the contact-dependent pathways including trogocytosis 27, tunnel nanotubes (TNTs) 42 and gap junctions 43, as well as the contact-independent extracellular vesicles 15 (**Figure 1**). Both microvesicles bud directly from the plasma membrane 1 and trogocytosis 44 are able to transfer membrane-associated Ags and functional p-MHCs presented on the cell surface, whereas exosomes derived from the late endosomes 45, 46, gap junctions 47 and TNTs 48 mainly transfer cytoplasmic Ag in the form of nucleic acid, Ag protein/peptide, and p-MHCs.

In fact, different modes of antigen transfer are involved in various physiological and pathological conditions. Trogocytosis is generally observed between cells with active membrane mobility. DCs trogocytose membrane fragments containing functional p-MHCs from neighboring cells are able to initiate immune response efficiently 49. Meanwhile, immunosuppressive molecules transferred to DCs via trogocytosis may lead to impaired immunity 50, 51. Gap junctions are hexamer channels formed within adjacent cells that facilitate the intracellular Ag exchange. For example, pathogenic and harmless antigen captured by gut-resident macrophages can be transferred to migratory DCs through gap junctions to induce protective immunity and establish oral tolerance, respectively 52-54. TNTs are actin-based membrane protrusions (up to 150 µm in length) that enable cell-to-cell connection over a longer distance. TNTs are the main mediators of lymphatic meshwork that support the quick activation of LN-resident DCs and promote the efficient induction of immune response 55. Likewise, TNTs formed with malignant cells or virus-infected cells may accelerate the spread of diseases 55, 56. EVs, on the other hand, enable a contact-independent Ag transfer between the donor and the acceptor. Tumor Ag transferred to DCs via EVs may consequently promote anti-tumor immunity or induce T-cell tolerance, depending mainly on the form of transferred Ag and the maturation state of receptor DCs 57-60.

### Trogocytosis

Generally, DCs phagocytize apoptotic and necrotic debris from the extracellular space for canonical Ag presentation and non-canonical Ag cross presentation 61-63. However, recent studies have demonstrated that DCs can also obtain antigenic information from living cells through a contact-dependent pathway called "trogocytosis" (also known as "nibbling", **Figure 1A**) 27, 40. Trogocytosis is an active process whereby acceptor cells conjugate to donor cells for extraction of surface molecules and membrane fragments 64. In the context of antigen transfer, membrane Ag and p-MHCs displayed on the surface of donor cells are transferred to DCs in close proximity via trogocytosis, which mainly involves close cell-to-cell contact, formation of “immunological synapse”-like structure, cross-cellular transport of plasma membrane-associated cargos, and separation of cells, leading to elicited immune responses or maintained peripheral tolerance 14, 65-67. Notably, the special biological characteristics of DCs facilitate the development of trogocytosis, including high membrane deformability and elasticity, rapid sensing and chemotaxis in respond to inflammation, and extensive interaction with other cells 10, 68. On the contrary, lines of evidence indicate that macrophages, which readily phagocytose apoptotic cells, cannot trogocytose membrane from viable cells, possibly due to limited expression of surface scavenger receptors 40, 44, too acidic endosomal/phagosomal environment, or high levels of lysosomal proteases 69.

In tumor-bearing patients, compared to apoptotic or necrotic tumor cells, live tumor cells expressing various tumor-associated antigens (TAAs) and tumor-specific antigens (TSAs) are the most abundant source of Ag with high immunogenicity. Therefore, trogocytosis of viable tumor cells by DCs contributes to an efficient and versatile Ag presentation for the activation of anti-tumor immune response 44. Meanwhile, during virus infection (e.g., human immunodeficiency virus (HIV) and EBV), DCs are able to preferentially acquire viral Ag from infected cells including lymphocytes, macrophages and non-hemopoietic cells, without risks of self-infection and immune dysfunction. On the other hand, DCs directly infected by virus may serve as Ag donors to provide sustained Ag for epidermal resident LCs or recruited circulating DCs 70-73.

However, attention should be paid to the fact that immunosuppressive molecules may also spread and spoil the immune microenvironment during trogocytosis. For example, human leukocyte antigen-G (HLA-G), a nonclassical HLA-class I molecule usually over-expressed by malignant cells, can directly inhibit the function, chemotaxis and viability of immune cells through receptor binding 74. Furthermore, the systemic immune environment can be further deteriorated when HLA-G is transferred to DCs via trogocytosis, which limits the activation of effector T cells, promotes the expansion of immunosuppressive cells (such as Tregs and myeloid-derived suppressive cells (MDSCs)), and even induces the apoptosis of immune cells, rendering tumor cells with greater metastatic potential 50, 51. Similarly, virus with high invasiveness and viability may also accelerate the speed and scale of transmission through antigen transfer. For example, although DCs are largely resistant to productive virus infection, they express high levels of C-type lectins, the main attachment factors of HIV at the surface of dermal and mucosal DCs. As a result, myeloid DCs, pDCs and LCs are all susceptible to infection with HIV, leading to impaired antigen-presenting function. In addition, follicular DCs (FDCs) capture large quantities of HIV as persistent reservoirs of virion to promote viral pathogenesis. Furthermore, HIV-pulsed DCs can transfer virion to T cells through “trans-infection” (across the virological synapse or DC-derived exosomes) and/or “cis-infection” (mediated by the *de novo* viral production within DCs) for facilitated viral dissemination and escaped antiviral immunity 75-77.

### Gap junctions

Gap junctions are clusters of intercellular hemichannels mainly composed of plasma membrane protein Connexin and formed in closely apposed neighboring cells 43 (**Figure 1B**), especially in DCs, B cells, monocytes and activated lymphocytes that have a high expression of Connexin 43 (Cx43) 78, 79. In such communication channels, ions and small molecules can be passively diffused 80. Moreover, gap junctions provide a pathway mediating the direct cell-to-cell transfer of Ag in the form of nucleic acids, proteins (molecular weights below 1 kDa, or amino acid residues less than 11) 81, 82, p-MHCs, and other signaling molecules 83. Of note, Cx43-based gap junctions are more favorable for the intercellular transfer of MHC I-restricted peptides with molecular weights lower than 1 kDa, instead of the theoretically larger MHC II-restricted peptides 62, 84.

Accumulating evidence suggests that gap junction plays an important role in the initiation and amplification of immune responses. It's reported that infection with bacteria *Salmonella* up-regulates the expression of Cx43 in both human and murine melanoma cells, which promotes the formation of functional gap junctions between melanoma cells and adjacent DCs to facilitate the intercellular transfer of antigenic peptides. Consequently, DCs present Ag on their surface to initiate specific cytotoxic T cells against tumor growth. Notably, such Cx43-dependent antigen transfer induces cross presentation and CD8^+^ T cell activation more efficient than that of standard Ag loading in generating anti-tumor responses 85, 86. Macrophages, although with limited capacity of Ag cross-presentation and CD8^+^ T cell activation, may serve as transfer stations of Ag to promote immune responses. Specifically, tumor rejection Ags are phagocytosed by macrophages 87 and subsequently transferred to DCs through gap junction-mediated intercellular transmission, which promotes the maturation of DCs and augments antitumor T cell responses 88. Such antigenic communication between macrophages and DCs can also be observed in the intestine. Mazzini et al. 47, 52 revealed that CX3CR1^+^ macrophages sampled over the intestine for suspicious “non-self” substances and delivered captured soluble Ags to DCs through gap junction. Subsequently, Ag-exposed DCs migrated toward the draining lymph nodes (dLNs) to prime or tolerize T cells, depending on the microenvironmental signals. FDCs have also been shown to form immune cell clusters with cognate follicular B cells by Cx43-mediated gap junction for direct Ag delivery 89, supporting the development and maturation of B cells in the germinal center 79.

### Tunnel nanotubes

Tunnel nanotubes (TNTs) (**Figure 1C**), also known as “filopodia bridges”, “membrane tubes” and “nanotubules” 90, are non-adherent, filamentous actin (F-actin) -based cytoplasmic protrusions 91 widely found in immune cells, neurons, tumor cells 56 and epithelial cells. TNTs enable cell-to-cell communication over long distance by plasma membrane bridges 92 (e.g., TNTs in macrophages can extend more than 150 μm 93), which establishes cytoplasm continuity 94 and facilitates intercellular information exchange. Specifically, nucleic acids, proteins, lipid nanoparticles, organelles (such as vesicles, lysosomes, mitochondria and autophagosomes) and even pathogenic particles 95 can be transported from donor cells to acceptor cells via TNTs 42. To date, “cell dislodgment" and "actin-driven" are the two widely recognized mechanisms accounting for the formation of intercellular TNTs 96. However, more efforts are needed to fully address the molecular basis and immunological significance of TNTs.

Despite insufficient understanding of TNTs-involved Ag transfer, lines of evidence suggest that such long and thin membrane tubes actively mobilize the immune regulatory networks by connecting multiple cells and promoting the intracellular sharing of antigenic information 97. It should be mentioned that the unique membrane structures of DCs including elaborate dendrites, sophisticated pseudopodia and delicate ruffles support the deformation and rearrangement of plasma membrane 98, 99, which also consists the structural basis of TNTs. Peripheral Ag-exposed DCs migrate to the dLNs within 48 h in a chemokine receptor 7 (CCR7)-dependent manner 14, 23, during which DCs undergo maturation with extensive dendritic stretching and remarkable morphological change, laying the foundation for immune cell communication and T cell activation 100. Then, LNs-resident DCs acquire Ag from their migratory counterparts by TNTs, which increases the availability of Ag and consequently magnifies immune response 1, 100. In addition, p-MHC class II complexes and costimulatory B7 family proteins (e.g., CD86 molecules) are shared within two adjacent B cells 101 or B cells and macrophages 102 through TNTs-mediated interconnection networks, which improves the efficiency of Ag-dependent T cell activation and induces a wide-ranging mobilization of the immune system.

Nevertheless, TNTs formed within tumor cells are reported to accelerate tumor metastasis by propagating metabolic plasticity, angiogenic ability and therapy resistance 56. Besides, TNTs can be exploited by pathogens such as HIV-1 for direct cell-to-cell spread 55.

### Extracellular Vesicles

Extracellular vesicles (EVs) (**Figure 1D**) are small spherical lipid bilayer particles released into the extracellular environment by almost all types of cells, including APCs, somatic cells and tumor cells. According to different mechanisms of biogenesis, EVs are mainly categorized into microvesicles (also named as microparticles) that bud directly from the plasma membrane 103 and exosomes secreted as a consequence of the fusion of multivesicular endosomes (MVEs) with the plasma membrane 104. EVs loaded with cargos (e.g., lipids, proteins and nucleic acids) are diffused into the interstitial space or the circulation to be internalized by receptor cells via phagocytosis, endocytosis, macropinocytosis, lipid rafts-mediated internalization, or direct plasma membrane fusion 105, 106. EVs remain attached to recipient cells can also transfer donor-derived cargos. For example, during allogenic organ transplantation, donor DCs migrate from the graft to lymphoid tissues and transfer MHC molecules to recipient cDCs through EVs. These EVs are internalized or remain attached to the recipient cDCs, instead of fusing with the plasma membrane of the acceptor APCs, which consequently enhanced the activation of alloreactive T cells. In this regard, depletion of recipient DCs after allograft can be used to delay graft rejection 107.

EVs-mediated Ag transfer from tumor cells or virus-infected cells to DCs is of great importance to the initiation and maintenance of specific immune responses 15. DCs are able to selectively engulf cancer cell-derived EVs incorporating antigenic protein, epitope peptide and/or p-MHCs through extracellular vesicles-internalizing receptors (EVIR) 45, 46, which coordinates antitumor response with quick mobilization and high efficiency 57. Of note, EVs can be easily isolated from the sera or malignant effusions of patient, representing as rich reservoirs of the whole panel of tumor Ag that may elicit a broad array of T cell clones against multiple Ag epitopes. Indeed, several EVs have been collected, modified and used as the next-generation cell-free cancer vaccines in personalized tumor immunotherapy 108, 109.

However, EVs with insufficient co-stimulatory signals and/or adjuvant-like components may induce immune tolerance when internalized by immature DCs 110. Moreover, immunosuppressive molecules can also be transferred through tumor cells-derived EVs 111-114 to impair the maturation and immunological function of immune cells.

## Location of antigen transfer

A growing number of studies have demonstrated that Ag is transferred at various physiological and pathological compartments that mainly include thymus 115, SLOs 1, intestine 47, allergic sites 116, allografts 117, lesions 34, 35 and vaccine injection sites 2, which largely determines the immunological consequence (i.e., immune activation or tolerance). And more efforts are needed to unveil other potential sites, as well as the associated outcomes, of antigen transfer.

### Thymus

Thymus is primarily responsible for the establishment of central tolerance that avoids autoimmune responses 118. Specifically, autoreactive T cells are negatively selected and eliminated in the thymic medulla before entering the periphery, which blocks the recognition of T cell receptors (TCRs) with tissue-restricted self-Ags and prevents specific cytotoxic killing against normal cells 30. Firstly, a subpopulation of medullary thymic epithelial cells (mTECs) displays the vast majority of autoantigens by generating corresponding p-MHCs, a process that involves the transcription factor autoimmune regulator (AIRE) 119, 120. Then, the resultant p-MHCs are subjected to other APCs in the medullary microenvironment such as DCs, B cells and macrophages, especially resident CD8α^+^ DCs, possibly through trogocytosis, exosomes and uptake of apoptotic bodies that are irrespective of the subcellular localization or expression pattern of Ag 121. As a result, medullary thymocytes that express TCRs with high affinity for autoantigen-associated p-MHCs presented by these APCs are either deleted through apoptosis or undergo lineage deviation that gives rise to Tregs and other 'unconventional' T cell populations 122. Notably, during negative selection, CD8^+^ and CD4^+^ single positive T cells may travel at a rate of 10 μm per minute in the medullary areas to increase the interaction with these APCs 123. It should be mentioned that scavenger receptor CD36 is involved in the process of antigen transfer from mTECs to DCs in the form of EVs that contain mTECs cell surface proteins (i.e., intact p-MHC class I and II complexes) 115. However, more efforts are needed to unveil the mechanistic details of such EVs-engaged antigenic communication and explore the participation of other antigen transfer approaches.

### Secondary lymphoid organs

SLOs, especially LNs and spleen, are highly organized structures that filter lymph and blood for suspicious Ags in these fluids, allow the entry of Ag-loaded DCs, and facilitate the antigenic interaction between DCs, B cells and T cells, serving as the “transit hubs” of adaptive immunity. There are a variety of specialized stromal cells, bone marrow cells and lymphocytes constituting the structural organization of SLOs for efficient Ag encounter and intercellular transfer. For example, LNs are anatomically composed of paracortex (T cell zone), cortex (B cell zone with follicles and germinal centers) and medulla (including subcapsular sinus (SCS), medullary sinuses, medullary cords and hilus) 124. These functionalized compartments are closely connected to orchestrate immune response against foreign substances.

Circulating DCs migrate back to the LNs upon peripheral Ag stimulation through afferent lymphatic vessels and in a chemokine-dependent manner for Ag transfer and lymphocyte activation 125, 126. Notably, the T cell immunity elicited within SLOs is found to be compartmentalized by route of lymphatic transport. In response to the administration of vaccinia virus (a replication-competent live attenuated vaccine), skin DCs fail to relocate to the dLNs from site of infection, which ablates vaccine efficacy 127. To delineate the underlying mechanisms, O'Melia et al. 128 designed a suite of nanoscale biomaterial tools to track and quantify the Ag access and presentation within LNs, thereby optimizing antitumor CD8^+^ T cell responses 121. They found that in the melanoma context, the extent of Ag presentation by dLNs-resident APCs remained unchanged despite the sustained access of lymph-draining Ag while the presentation of cell-transported Ag was increased, which was partially caused by the phenotypes of DCs accessed via different lymphatic transport mechanisms. Specifically, passively drained Ag was presented mainly by pDCs and cDCs that displayed an immunosuppressive phenotype. In contrast, actively transported Ag was presented by dDCs and LCs that exhibited an immunepotentiating phenotype. However, the complex communication among different cells, especially the intercellular antigen transfer, is still incompletely understood. More detailed discussion of intra-SLOs antigen transfer can be found at the following chapters (i.e., 5.1 From APCs to APCs).

Generally, antigen can be transferred within the SLOs in multiple forms, including Ag protein/peptide, Ag-encoding nucleic acid, functional p-MHC, immune complex, and vaccine particle. More importantly, the form of Ag may affect the mode and even the immunological consequence of Ag transfer. In SLOs, LNs in particular, exogenous Ag or Ag fragments (i.e., antigenic complexes, protein and peptide) transferred to DCs by trogocytosis, EVs, TNTs or gap junctions can be canonically presented on the MHC class II molecules to activate specific CD4^+^ T cells 3, 62, 129 or cross-presented via the MHC class I molecule-restricted pathway to initiate specific CD8^+^ T cells 63. Meanwhile, Ag-encoding nucleic acids (e.g., mRNA, pDNA) transferred to DCs, probably through EVs, TNTs or gap junctions, can be translated into “endogenous Ag” and then preferentially presented on the MHC class I molecules or undergo Ag translocation to the endosomes for MHC class II-favored cross presentation 6, 7, 130, 131. Besides, functional p-MHC I and/or p-MHC II can be transferred to DCs mainly through trogocytosis and EVs, which facilitates an efficient elicitation and magnification of T-cell responses 49, 132-134.

### Intestine

**Figure 2:**
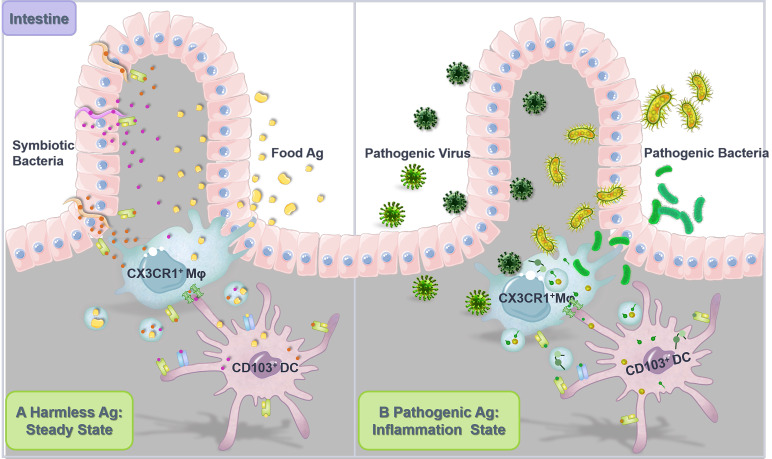
**Antigen transfer in the intestine. (A)** Intercellular transfer of harmless Ag establishes intestinal homeostasis. Gut-resident CX3CR1^+^ macrophages (Mφ) continuously sample the gut lumen for harmless soluble Ag, including Ag from dietary proteins and commensal bacteria. Subsequently, Mφ captured Ag is transferred to intestinal migratory CD103^+^ DCs, which then migrate back to the dLNs to induce T cell tolerance, establishing intestinal flora homeostasis and preventing food allergy. **(B)** Intercellular transfer of pathogenic Ag induces pro-inflammatory responses against infection. Upon intestinal invasion of pathogenic bacteria and viruses, CX3CR1^+^ Mφ collect potentially pathogenic Ag from infected intestinal tissue cells or directly from the pathogen, which was further transferred to CD103^+^ DCs through gap junctions and EVs for presentation and T cell activation, inducing specific protective immune responses.

Chronically exposed to both innocuous and pathogenic Ags, intestine constitutes the largest and most complex part of the immune system where acquired oral tolerance to harmless dietary proteins and commensal bacteria is established while specific immune response against pathogenic microbes can be elicited 135. It is increasingly recognized that in the intestine, antigen transfer among phagocytes with specialized functions 136 plays a vital role in mediating the balance between tolerance (**Figure 2A**) and protective immunity (**Figure 2B**).

Intestinal APCs, especially DCs, are in dispensable for triggering peripheral Foxp3^+^ Tregs polarization from naïve T cells and inducing oral tolerance 137, 138. And default responses to harmless Ags may otherwise lead to food allergies, inflammatory bowel disease, and even colorectal cancer 139, 140. Mazzini et al. 52 found that soluble food Ags are internalized by gut-resident CX3CR1^+^ macrophages and quickly transferred to migratory CD103^+^ DCs in a Cx43-dependent and plasma membrane-required manner (i.e., through gap junction), which consequently promoted Treg differentiation and induced oral tolerance to these Ags. Meanwhile, McDole et al. 141 suggested that in steady state, goblet cells in the epithelium of small intestine transported low molecular weight soluble Ags from the intestinal lumen to tolerogenic CD103^+^ DCs in the lamina propria to promote intestinal immune homeostasis. However, the underlying mechanisms accounting for such Ag transfer from goblet cells to DCs remain to be fully elucidated. Segmented filamentous bacteria (SFB) and other intestinal resident commensal bacteria adhere tightly to intestinal epithelial cells (IECs) via hook-like structures, and Ag proteins from these bacteria can be transferred into the cytosol of IECs through adhesion-directed endocytosis to affect host T cell homeostasis 142. Specifically, at the tip of the SFB-IEC synapse, SFB generates endocytic vesicles containing microbial cell wall-associated proteins, including an Ag that induces mucosal T helper type 17 (Th17) cell differentiation, to be acquired by host IECs for elicitation of specific T cell responses.

On the other hand, intercellular antigen transfer might also occur in the context of gastrointestinal infections that consequently induces protective immune defense against potentially pathogenic Ags 53, 54. In the rectal mucosal biopsies of patients with acute campylobacter colitis or cholera, mononuclear phagocytic cells (mainly macrophages and DCs) in the superficial rectal mucosa exhibit a higher prevalence of ultrastructural features of activation. Macrophages are found to actively insert pseudopodia through intestinal epithelial cell gaps to capture pathogenic Ag, while DCs that are superior in Ag presentation and T cell activation display active membrane processes, enhanced macropinocytosis and elevated phagosomal/lysosomal activity 143, indicating that macrophages and DCs might share antigenic information within the intestine through multiple pathways to coordinate the anti-infection immune responses.

### Allergic sites

Allergy, also termed as allergic disease or anaphylactic reaction, refers to hypersensitivity of the immune system in response to the exposure of typically harmless Ags. To date, mounting evidences have suggested that intercellular transfer of immunoreactive substance or Ag is closely associated with the development of exaggerated immune response to allergens such as pollens, dust mites, furry animal dander, drugs and foods.

Mast cells (MCs) are well recognized as key effector cells of allergic reactions, which respond to endogenous or exogenous danger signals by secreting a plethora of mediators including histamine, proteases and cytokines in the form of mast cell granules (MCGs) that can be released by degranulation within seconds on activation to initiate immune responses, neutrophil recruitment and allergen clearance. On skin inflammation, MCs-exocytosed intact MCGs are engulfed by and degraded within dermal DCs to promote DC maturation and migration to the dLNs for subsequent T cell priming 144. In turn, it is reported that CD301b^+^ perivascular DCs continuously sample the blood and relay Ag to neighboring MCs and other DCs through an active discharge of surface-associated Ags on microvesicles (MVs) generated by vacuolar protein sorting 4 (VPS4) to potentiate inflammation and anaphylaxis against blood-borne Ags 115. Moreover, in the case of allergic asthma and atopic dermatitis (AD), the interplay between tissue structural cells and DCs is largely responsible for CD4^+^ T helper type 2 (Th2) cell-induced dysregulated type 2 inflammation (Th2 sensitization) to environmental allergens 145. For instance, when exposed to house dust mite (HDM), airway epithelial cells generate danger-associated molecular patterns (DAMPs), chemokines and cytokines to recruit, activate and skew DCs toward Th2 phenotype that promotes the pulmonary inflammatory reactions. Whereas skin KCs recognize HDM through Toll-like receptors (TLRs) and produce type 2 immune cytokines to activate cDC2 subsets and induce their migration to the dLNs for elicitation of Th2 response. Moreover, individuals with autoimmune diseases, such as systemic lupus erythematosus (SLE) that produces systemic inflammation in multiple organs, have platelets that continuously recruit and release mitochondrial DNA (mtDNA) as a source of circulating autoantigen to exacerbate the self-attack of immune system 146.

### Allografts

Similar to that of allergy, the rapid acquisition of antigenic information from allograft by host APCs induces severe immune rejection and graft organ necrosis. During allogeneic organ transplantation, host DCs rapidly integrate intact donor p-MHC class I complexes through cross dressing or uptake and process donor Ags into allopeptides bound to self-MHC molecules, which induces massive proliferation of reactive T cells and leads to graft rejection 147, 148. In addition, donor DCs migrated from the graft to the SLOs may release EVs to facilitate an efficient passage of donor MHC molecules to host cDCs, which triggers full activation of alloreactive T cells and impedes graft survival 107. On the other hand, DCs in successfully transplanted patients undergo continuous transfer of p-MHCs from donor DCs and/or donor somatic cells to DCs, and these MHC-dressed DCs may induce immune tolerance to benefit a long-term graft survival by upregulating their own programmed death-ligand 1 (PD-L1) 149.

In order to minimize the Ag transfer-associated graft rejection, Borges et al. 150 incubated skin grafts with the anti-inflammatory mycobacterial protein DnaK, which promoted a March 1-dependent reduction of MHC class II molecules on donor CD103^+^ DCs, thereby inhibiting the transfer of p-MHCs to recipient DCs and prolonging the survival of transplanted skin. Meanwhile, Zhang et al. 151 used CRISPR/Cas9 to ablate costimulatory CD40 at the genomic level in DCs dressed with donor p-MHCs to inhibit their maturation and LNs-homing, which not only induced long-term graft tolerance but also prevented severe immunosuppressive side effects.

### Pathological lesions

Antigen transfer at the lesions (e.g., sites under physical damage, chemical stimulation, ultraviolet irradiation, pathogen infection and tumorigenesis) may serve as a critical line of immune defense. For example, in human skin models and genital herpes lesion biopsies, HSV is first taken up by LCs that patrol over the epidermis. Subsequently, HSV-loaded LCs migrate to the dermis and transfer HSV Ag to CD103^+^ cDCs with a superior antigen-presenting ability and more motivated LNs-homing for initiation of immune response (passive Ag transfer, as HSV-infected LCs undergo apoptosis to be further taken up by dermal DCs) 152, 153. Notably, cDC1s that feature high expression of C-type lectin-like receptor 9A (CLEC9A) are capable of binding dead-cell debris and promoting the cross presentation of corpse-associated Ags, which facilitates their relay of Ag from the donor cells or directly from the pathogens 154, 155. Moreover, during skin inflammation, an intensive and long-lasting synapse-like contact between migratory DCs and stationary MCs culminates in the functional transfer of DC-restricted proteins to MCs, including MHC class II complexes, which may ensure the host defense during DC migration to the dLNs or critical periods of migration-based DC absence 156. In the context of tumorigenesis, p-MHC class I complexes and other membrane structures containing the “non-self” Ags that presented on the surface of tumor cells can be directly transferred to DCs via trogocytosis 44, while intracellular Ags can be transmitted to DCs through exosomes 157. Squadrit et al. 57 reported a lentivirus-encoded chimeric receptor named extracellular vesicle-internalizing receptor (EVIR) to facilitate the specific and efficient uptake of cancer cell-derived EVs by DCs, which exploited the cross dressing of pre-formed p-MHC class I complexes for improved activation of specific T cell responses against tumor.

However, as aforementioned, some immunosuppressive molecules might also be transferred to immune cells through trogocytosis, gap junctions, TNTs and EVs to modulate immune responses and promote disease progression 50, 51, 74. For example, natural killer (NK) cells acquire carcinoembryonic antigen (CEA) from the surface of CEA-expressing cells via trogocytosis and exhibit inhibited cytolytic activity and dampened degranulation function 158; T cells exposed to tumor-derived exosomes incorporating PD-L1 display suppressed activation in the dLNs 159; and TNT-connected astrocytoma cells may promote tumor progression and resistance to therapy 160.

### Vaccine injection sites

Prophylactic and therapeutic vaccines are generally administrated into the intramuscular, subcutaneous or intradermal compartments. Different physiological sites differ in the cell type, cell abundance and lymphatic system. Therefore, the site of vaccine inoculation may affect the efficacy of Ag transfer as well as the strength and duration of immune response 2.

For example, upon intramuscularly injection, self-amplifying mRNAs (SAM^®^)-encoded Ag is expressed by muscle cells and then transferred to nearby APCs, which consequently promotes the activation the CD8^+^ T-cell responses 33. In addition, mRNA-based vaccine is taken up by both immune and non-immune cells in the skin upon intradermal administration 32. Functional Ag may be expressed by these vaccinated non-immune cells and then transferred to APCs to promote the induction of adaptive immunity. Moreover, studies suggest that many keratinocyte-specific molecules can be transferred to epidermal-resident LCs as mRNA and protein probably via TNTs or dendrites 161, 162.

Administrated vaccine antigen internalized by non-APCs at the injection site can be transferred to tissue-resident APCs or migratory DCs. Meanwhile, migratory DCs may migrate towards the draining SLOs and transfer both directly captured and indirectly acquired (transferred from non-APCs) Ag to LN-resident or splenic DCs. Detailed information will be discussed in the following sections.

## Participant of antigen transfer

The phenomenon of antigen transfer was initially identified in T cell activation. In fact, within minutes of cognate T cells interacting with APCs, p-MHCs on the surface of APCs form clusters at the site of T cell contact. Subsequently, clusters containing p-MHCs are internalized by T cells via TCR-mediated trogocytosis. As a result, T cells are subjected to the Ag-specific cytolysis by neighboring T cells (termed “fratricide”), which may lead to suppressed T cell immunity 163. Meanwhile, T cells may also acquire p-MHCs from other target cells through contact-dependent immunological synapses, and Tregs are especially adept at removing MHC class II and costimulatory molecules from APCs via trogocytosis to induce immune tolerance 164. In addition to T cell-based Ag receptors, NK cells 165, 166 and basophils 167 can also acquire Ag from APCs, thereby impacting the potency, durability, and even consequence of immune responses.

Generally, antigen transfer is a reciprocal interaction that theoretically can occur between any cells with active membrane mobility, including that from APCs to APCs, from non-APCs to APCs, from APCs to non-APCs, and even from non-APCs to non-APCs. In this review, we focus on DCs-based Ag receptors and the associated immunological outcomes (**Table 1**).

### From APCs to APCs

**Figure 3:**
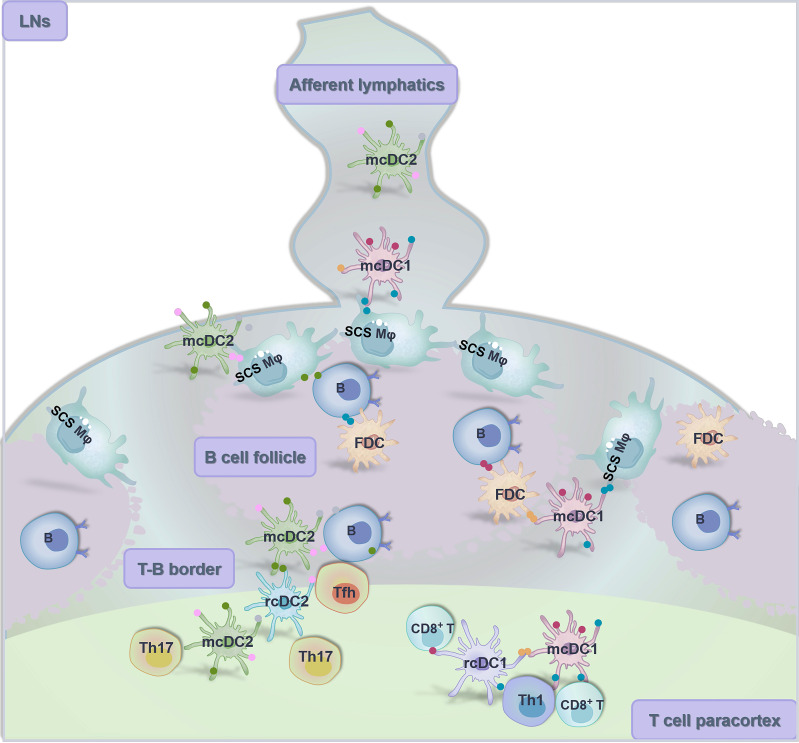
**Close intercommunication among B cells, macrophages and DCs in the LNs.** Peripheral migratory conventional DC1 and DC2 (i.e., mcDC1 and mcDC2) move back to the draining LNs via afferent lymphatics and transfer Ag (including viruses, particulate Ag and immune complexes) to CD169^+^ subcapsular sinus (SCS) macrophages that line the follicle-proximal side of the SCS. Then, these SCS macrophages display Ag to follicular B cells via cellular protrusions, and follicular DCs (FDCs) located therein engage in reciprocal Ag sharing with B cells for elicitation of germinal center reactions. Afterwards, mcDC1 and mcDC2 pass through the T-B boundary and enter the T cell-localized medullary zone to share Ag with resident conventional DCs (rcDCs) for efficient activation of immune responses.

It is widely recognized that DCs, especially cDCs, are indispensable coordinators of the adaptive immunity, yet elicitation of specific immune response may not rely solely on the direct antigenic stimulation on DCs. Accumulating evidence suggests that Ag or Ag complex can be transferred from other types or individuals of APCs to DCs 168 (**Figure 3**), contributing to an improved availability of Ag that mobilizes the immune system with higher efficiency.

#### DCs and DCs

S.L. Nutt et al. 79 have summarized that heterogeneous DCs subpopulations are closely associated with each other in the systemic immune network despite distinct developmental, locational, phenotypical and functional hallmarks, which constitutes the immunological basis of T cell activation and tolerance 169, 170.

It is reported that in response to CD40L-expressing Th cells or recombinant CD40L, networks of TNTs are induced by DC1 (i.e., DCs matured in the presence of inflammatory mediators of type-1 immunity) to support the direct intercellular transfer of endosome-associated vesicles and Ag between DCs 55. Aline et al. 171 demonstrated that DCs-derived exosomes encompassing functional MHC class I/II and costimulatory molecules were capable of inducing protective immunity against toxoplasmosis, serving as a novel cell-free vaccine. Specifically, part of the adoptively transferred *Toxoplasma gondii*-pulsed DC-derived exosomes accumulated in the spleen and were most likely internalized by spleen-resident CD8α^+^ DCs, which elicited a strong systemic T helper type 1 (Th1)-biased specific immune response. In addition, protein antigens in DCs-derived exosomes can be transferred to and presented by recipient DCs to induce the activation of allogeneic T cells, which may be used to facilitate cancer immunotherapy 172, 173. On the other hand, inflammatory signals induce the LNs-homing of migratory cDC1 and its subsequent Ag transfer to LNs-resident DCs through tight synaptic interaction 15, which facilitates the accumulation of Ag in LNs-resident DCs for activation of specific effector CD8^+^ T cells 1. pDCs, formerly known as natural interferon producing cells (NIPCs), are the main producers of type I Interferon (IFN) 18 and play a key role in antiviral immunity. Although the capability of pDCs to generate *in vivo* cross-primed CD8^+^ T cells remains controversial, they have been shown to transfer antigen (possibly Ag protein, peptide, or p-MHC I 133) to the bystander cDCs via EVs, which leads to efficient cross-priming of naive CD8^+^ T cells and induction of durable immunity. Notably, although both cDC1s and cDC2s are capable of acquiring Ag from pDCs, cDC1s, instead of cDC2s, are required for CTL activation upon pDCs-targeted vaccination 132. Furthermore, monocytes loaded with protein or peptide antigen can transfer Ag to splenic DCs through cell-cell contact and the formation of Cx43-containing gap junctions, which leads to efficient activation of CTLs and potent antitumor responses 174.

#### Macrophages and DCs

With intricated membrane structures and dynamic membrane activities, macrophages and DCs are closely associated in the context of antigen transfer (**Figure 2**). Although macrophages prevail in phagocytosis, their ability of Ag cross presentation is far inferior than that of DCs. However, studies suggest that there exists a complicated interplay between macrophages and DCs in the process and presentation of Ag. For instance, upon dead cell accumulation *in vivo*, macrophages transfer phagocytosed Ag to DCs via exosomes for potent antigen presentation and efficient T‐cell activation 181. In addition, despite inefficient cellular uptake of Listeria monocytogenes (Lm), DCs are capable of taking up microparticles (MPs) released by Lm-infected macrophages. These MPs transport Lm Ag to DCs for presentation, propagating DC-elicited protective immunity against Lm infection 175. Similarly, macrophages act as transmitters to convey Ag for presentation by DCs in response to the invasion of other pathogens such as mycobacterium 176, 177 and salmonella 178. Moreover, it is found that infected macrophages secrete EVs containing Cdc42 (a protein responsible for increased cellular endocytic activity) to enhance the cellular uptake of recipient cells 179, which may further facilitate the antigenic cross-talk between macrophages and DCs.

#### B cells, macrophages and DCs

A successful elicitation of the humoral immunity depends primarily on the close antigenic interaction among B cells, macrophages and DCs. In both LNs and spleen, the maturation and native antigen presentation of B cells requires the support from follicular dendritic cells (FDCs) and CD169^+^ subcapsular sinus (SCS) macrophages 180. Specifically, at the T-B border, SCS macrophages display Ag including processed viral particles, vaccine particles and immune complexes 181, 182 to both cognate and non-cognate B cells via TNTs-like cellular protrusions that extend into follicles. SCS macrophages-mediated Ag recognition by cognate B cells through B cell antigen receptors (BCRs) initiates the early activation and the subsequent migration to T-B border of B cells 183. Meanwhile, immune complexes are transferred from SCS macrophages to non-cognate B cells, and then ferried into the follicle for deposition on FDCs 183, 184. Then, FDCs retain native Ag for prolonged presentation to B cells that evokes the germinal center reactions and promotes the maturation of effector and memory B cells. Notably, evidence suggests that in the lymphoid germinal center, direct intercellular communication through gap junctions is involved in FDCs-FDCs and FDCs-B cells interaction, in which multiple signal molecules and Ag fragments/complexes can be shared 89. On the other hand, there is mounting evidence that both migratory and resident cDCs may encounter cognate B cells at the T-B border and contribute to their early initiation 180, 185. Besides, Lectin-like oxidized low-density lipoprotein receptor-1 (LOX-1) signaling on DCs promotes B cell differentiation into class-switched plasmablasts and facilitates their exit from germinal center and migration towards local mucosa and skin 186. Furthermore, result illustrates that Ag acquired by B cells through BCRs can be specifically transferred to B220^+^ macrophages through direct cell-cell contact, which enables the macrophages to activate CD4^+^ T cells 187.

### From non-APCs to APCs

Given the homologous expression of MHC class I molecules by all nucleated cells, non-APCs can also serve as Ag donor cells to APCs, especially to DCs, including malignant/transformed cells, vaccinated muscle cells and KCs, and even harmless commensal bacteria. Antigen transfer from non-APCs to APCs may promote the immune response against the “non-self” or facilitate the spread of invasive pathogens.

#### Tumor cells and DCs

**Figure 4:**
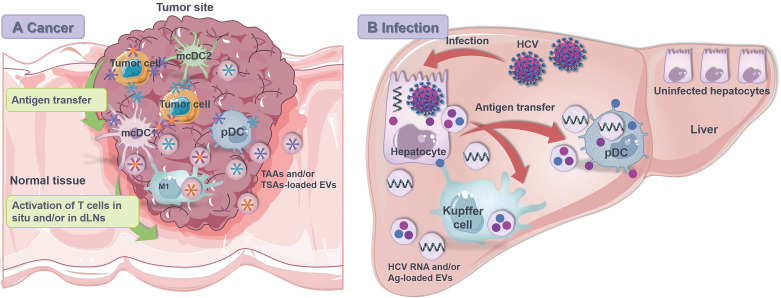
**Antigen transfer from non-APCs to APCs in the context of anti-cancer/-infection immunity. (A)** Tumor cells serve as the largest Ag reservoir for migratory conventional DCs (mcDCs), plasmacytoid DCs (pDCs) and macrophages. Ag is transferred from tumor cells to these APCs through both contact dependent (i.e., trogocytosis, gap junctions and TNTs) and independent (i.e., EVs) pathways, leading to activation and expansion of T cells *in situ* or in the dLNs. **(B)** Hepatitis C virus (HCV)-infected parenchymal hepatocytes release virus RNA and protein Ag through both contact dependent and independent pathways for activation of Kupffer cells and pDCs against virus invasion. TAAs, tumor associated antigens; TSAs, tumor specific antigens.

Tumor infiltrating DCs assume different functional states that affect the antigen transfer and overall antitumor immunity (**Figure 4A**). In the TME, interferon regulatory factor 8 (IRF8)-dependent CD103^+^ cDC1 (CD141^+^ cDC1 in human) are the only APCs that can cross present tumor rejection Ags for activation of specific CTLs 23, which are sparsely distributed and frequently threatened by the hostile immunosuppressive environment 188. In this situation, antigen transfer, especially cross dressing (i.e., p-MHCs transfer), from tumor cells to cDC1s stands as an efficient means of Ag presentation and reactive T cell activation 44, 189. On the other hand, cDC2 is developmentally driven by interferon regulatory factor 4 (IRF4) and highly specialized in MHC II-restricted presentation 29. Recently, Duong et al. 190 investigated the transcriptional profiles of intra-tumoral DCs within regressor tumors and identified an activation state of CD11b^+^ cDC2 with interferon-stimulated gene signatures. Stimulated by exogenous IFN-β, these cDC2 acquired and presented intact tumor-derived p-MHC class I complexes to induce CD8^+^ T cell-involved antitumor immunity against progressor tumors in mice lacking cDC1 188, 190. Moreover, Bonaccorsi et al. 191 identified that pDCs, although inefficient in internalizing cell membrane fragments by phagocytosis, were able to acquire membrane patches and associated molecules from cancer cells of different histotypes in a cell-to-cell contact-dependent manner that closely resembled “trogocytosis”. As a result, tumor cell-derived Ag was displayed by pDCs and recognized by specific CD8^+^ T cells to promote anti-tumor cellular immune response.

#### Virus infected cells and DCs

During hepatitis C virus (HCV) infection, exosomes mediate the intercellular transfer of immunostimulatory HCV RNA from infected cells to neighboring non-infectible pDCs to trigger the generation of type I IFN 192 (**Figure 4B**). Both HCV-infected cells and purified HCV RNA-packaged exosomes are sufficient to activate pDCs without infecting them. Notably, the exosomal viral RNA transfer is dependent on active viral replication, direct cell-cell contact and TLR 7 signaling 193. Nevertheless, such exosome-mediated transfer of viral RNA may enhance virus clearance by activating Kupffer cells and pDCs or promote virus infection by delivering infectious viral genomes to cells that are permissive for viral replication.

#### Vaccinated somatic cells and DCs

To improve the efficacy of protein- or nucleic acid- based vaccines, substantial efforts have been paid to promote the site-specific accumulation of vaccine components in SLOs, and even in APCs 194-199, which increases the availability of vaccine Ag by DCs to amplify specific immune response and establish a durable memory. However, a targeted delivery of vaccine preparation proposes great demands for its physiochemical properties (e.g., particle size, potential and surface modification) and route of administration 200, 201. Moreover, compared to professional APCs that have a limited cell abundance in different vaccination sites, somatic cells with larger quantity and widespread distribution display higher competence in messenger RNA (mRNA)-transfection and protein-uptake, which may impact the magnitude and duration of specific immunoresponse by transferring Ag to nearby APCs (specific mechanisms of Ag transfer need to be further identified) 2. Indeed, most somatic cells are biologically equipped with abundant cytoplasmic free ribosomes (such as KCs and muscle cells) or rough endoplasmic reticulum-attached ribosomes (such as hepatocytes and fibroblasts) to support their antigenic communication with surrounding DCs 33. For example, KCs actively transfer Ag, including Ag-encoding mRNA 161 and protein 162, to the skin-resident LCs mainly in a contact-dependent fashion, impacting the efficacy and safety of transdermal- and intramuscular- injected vaccines.

#### Commensal bacteria and DCs

It is reported that several bacteria participate in tumor immunosurveillance and antitumor immune response. Rong et al. 202 studied the bacteria-reactive CD8^+^ T cell response in HBV-associated hepatocellular carcinoma patients and found that circulating CD8^+^ T cells displayed remarkable enhanced immune responses against a series of commensals and bacteria, including Escherichia coli (E. coli), Enterococcus faecium, Bifidobacterium longum, Bacteroides fragilis, and Enterococcus hirae. And the ratio of CD8^+^ T cell-to-Foxp3^+^ Treg was positively correlated with the proportion of Bifidobacterium longum-reactive and Enterococcus hirae-specific CD8^+^ T cells, whereas the frequency of PD-1^+^ CD8^+^ T cells was negatively correlated with the frequency of Enterococcus hirae-specific CD8^+^ T cells. Moreover, these bacteria-reactive responses were MHC class I-restricted and dependent on the presence of APCs, indicating that certain commensal bacteria might act as Ag mediators between cancer cells and APCs to increase the proportion and viability of tumor-reactive IFNγ^+^ T cells 202, which is also observed in MC38 colon cancer, MCA-205 sarcoma and RET melanoma 203-205.

## Vaccine effect of antigen transfer: immune amplification or tolerance

Antigen transfer plays an important role in coordinating immune amplification and tolerance. When the receptor cells are tolerogenic DCs, immature DCs, some pDCs and even certain types of non-APCs 110, 138, 165, 206-208, antigen transfer may promote the expansion of immunosuppressive Tregs/MDSCs and even induce the apoptosis of specific T cells, leading to tolerance 47. Immune tolerance is fundamental to the maintenance of homeostasis. For example, antigen transfer from mTECs to DCs in the thymus enables the deletion of self-reactive T cells and promotes the establishment of central tolerance 118-120. In patients with autoimmune diseases, harmless Ag (e.g., autoantigens and dietary proteins) are recognized as pathogenic Ag, which consequently causes local/systemic inflammatory responses that are harmful and even fatal. In this situation, antigen transfer that induces tolerance to specific Ag may limit autoimmune responses and help restore homeostasis 146, 209, 210. On the other hand, antigen transfer to mature APCs, especially DCs, may facilitate the access and presentation of Ag that contributes to a more efficient and versatile elicitation of the adaptive immunity 1, 48, 55, 134, 190, 211, which is frequently used to enhance the preventive and therapeutic effects of vaccines 26.

Vaccines are powerful weapons against pathogenic evasion 26 and tumor progression 212-216, which elicit specific T/B lymphocyte-mediated effector and memory immune responses upon single or repeated inoculation. Considering the efficacy and biosafety, most recently licensed vaccines are typically protein/peptide-based subunit vaccines that usually used in combination with adjuvants, nucleic acid-based vaccines (especially mRNA vaccine) 217-219, and DCs-based vaccines 220, 221. And transcutaneous local injection is the most applied route of administration for these vaccines 222, including:

**1) Intradermal injection (i.d.)**, which is most frequently used for the inoculation of bacillus Calmette-Guérin (BCG), rabies and smallpox vaccines 223 for its little invasiveness, avoided drug degradation in the gastrointestinal tract, and escaped hepatic first-pass effect. Vertebrate skin comprises epidermis and dermis. Epidermis is composed of abundant KCs and few melanocytes and LCs. In contrast, dermis is rich in collagen and elastin fibers but low in cell density. Dermal APCs (such as cDCs, mo-DCs, LCs and macrophages) 224 and lymphatic system facilitate a quick and effective initiation of immune response, conferring dermis a highly immunocompetent site for vaccine delivery 225, 226.

**2) Subcutaneous injection (s.c.)**, that is most suitable for the administration of live-attenuated vaccines against polio, measles, mumps, rubella and yellow fever. Subcutaneous compartments incorporate blood vessels, nerves, loose connective tissue and adipose tissues, where fibroblast, mast cell and macrophage are most abundant. Subcutaneous drainage system is underdeveloped, which prolongs the in-situ Ag dwelling and serves as Ag reservoirs.

**3) Intramuscular injection (i.m.)**. As the most commonly used route of delivery for licensed vaccine, especially inactivated vaccines against hepatitis A/B (HepA/B), HPV, influenza, and the currently prevalent severe acute respiratory syndrome coronavirus 2 (SARS-CoV-2), i.m. is easy to perform and generally well tolerated, with a low risk for adverse reactions 212-214, 227. Muscle tissue is composed primarily of myocytes, with few APCs, blood vessels and nerves. Therefore, higher dosage, adjuvant-incorporation, and multiple administration is usually recommended for eliciting an expected immunoprotection 228.

In addition, intravenous (i.v.) and intranodal (i.n.) injection have also been studied 229. However, their feasibility and safety needs to be further optimized before putting into clinical use 230. It's worth noting that a long-term persistence of immunogens/immunomodulators or sustained expression of vaccine products was observed at the site of delivery following i.d., s.c., and i.m. (superficial injection). Meanwhile, upon i.v., i.m. (deep injection), and intraperitoneal injection (i.p.), significant antigenic signal was detected in the liver early post administration 231, 232, suggesting that the biodistribution of vaccine is route-dependent, and liver may be an important anatomical compartment for mounting immunoreactions.

**Figure 5:**
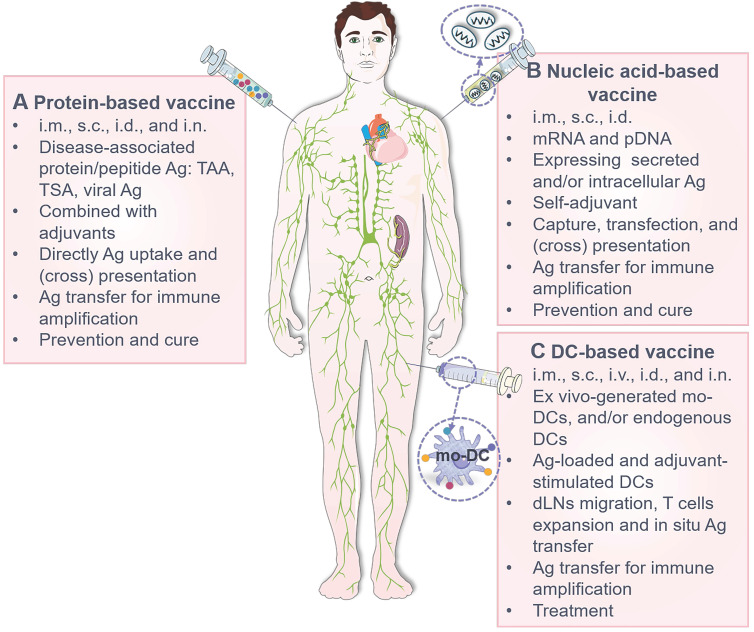
**Characteristics of the currently most licensed three vaccine types**. **(A)** Protein-based vaccines. Widely used in clinical practice, these vaccines incorporate disease-associated Ag protein/peptide (usually insufficient in immunogenicity) and adjuvants (such as alum and Freund's adjuvants) and are mainly administrated via i.m., s.c., i.d., and i.n. (less adopted). After administration, Ag is transferred from vaccinated somatic cells (such as muscle cells, keratinocytes and fibroblasts) and APCs (such as rDCs, LCs and macrophages) to DCs at the injection site to amplify immune responses for disease prevention and treatment. **(B)** Nucleic acid-based vaccines. These vaccines contain Ag-encoding mRNA or plasmid DNA (pDNA) with self-adjuvant effects and are inoculated mainly by i.d., s.c. and i.m. After injection, vaccine particles/naked nucleic acids are internalized, translated, processed and presented by local somatic cells and APCs, or undergo Ag (the original Ag, translated/process/displayed Ag fragments or Ag complexes) transfer to DCs, serving as prophylactic and therapeutic agents. **(C)** DC-based vaccines. Primarily administrated through i.d., s.c., i.m., i.v. and i.n. (less adopted), these vaccines are mainly composed of *ex vivo*-cultured mo-DCs derived from autologous/allogeneic mononuclear progenitor cells or endogenous cDCs/pDCs isolated and enriched from blood to provide an individualized therapeutic effect. These DCs are pulsed with Ag and adjuvant prior to administration, and Ag-laden DCs can also transfer Ag to nearby APCs *in vivo*. i.d.: intradermal injection; i.m.: intramuscular injection; i.n.: intranodal injection; i.v.: intravenous injection; s.c.: subcutaneous injection; TAA: tumor associated antigen; TSA: tumor specific antigen.

Antigen transfer takes place after vaccine inoculation, which is primarily grouped into the following categories according to the type and tissue distribution of vaccine (**Table 2**): **1)** Antigen (such as Ag-encoding nucleic acids, Ag peptides/fragments, intact Ag proteins, particulate Ag, immune complex and functional p-MHCs) transfer from vaccinated APCs and/or non-APCs to neighboring DCs at site of administration in the context of protein/peptide-based vaccines (**Figure 5A**) and nucleic acid-based vaccines (**Figure 5B**); **2)** Antigen transfer from Ag-pulsed DCs to nearby APCs including LCs, cDCs and macrophages at vaccine inoculation site in terms of DCs-based vaccines (**Figure 5C**); and **3)** Antigen sharing from Ag-laden DCs to SCS macrophages, B cells, FDCs and cDCs at the dLNs to activate germinal center reactions.

### Protein-based vaccines

**Figure 6:**
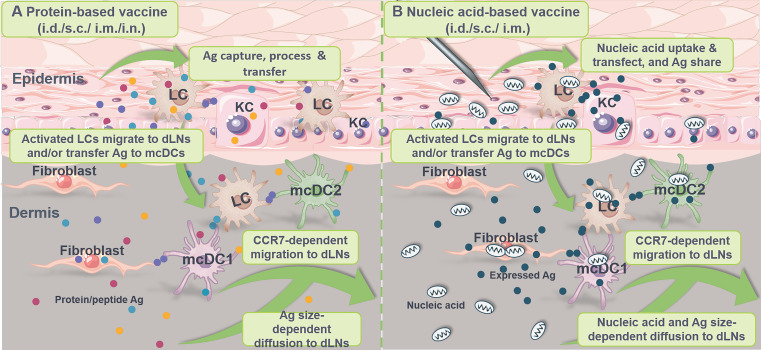
**Antigen transfer in protein-based vaccines and nucleic acid-based vaccines. (A)** After local administration of protein-based vaccines, Ag is captured, processed, presented and/or intercellularly shared by epidermal LCs and keratinocytes (KCs). Subsequently, activated Langerhans cells (LCs) migrate to the dermis for activation of migratory conventional DCs (mcDC1 and mcDC2) via antigen transfer. Meanwhile, fibroblasts in the dermis may also internalized and transfer Ag to DCs. Activated dermal cDCs and LCs then homing to the dLNs in a CCR7-dependent manner to induce immune response. Meanwhile, free Ag particles may also diffuse into the dLNs in a size-dependent manner to directly activate adaptive immunity. **(B)** Nucleic acid-based vaccines are locally administrated to be internalized and transfected by LCs, KCs and fibroblasts. Subsequently, these vaccinated cells may transfer Ag to skin DCs for dLNs-homing and immune activation. Similarly, nucleic acids and expressed Ag may directly drain toward the dLNs to induce immune response. i.d., intradermal injection; s.c., subcutaneous injection; i.m., intramuscular injection; i.n., intranodal injection.

Increasing studies suggest that the efficiency of T cell-mediated adaptive immunity against peripheral infections and particulate vaccine systems (such as nanoparticles, microparticles and adjuvant-formulated proteins) depends heavily on the ability of LNs-homing and Ag presentation by peripheral DCs 233. In addition, it seems that most soluble Ags cannot penetrate into the paracortex and cortex of LNs, which directly limits the Ag accessibility of LNs-resident DCs 237, 238. At the same time, anatomic studies indicate that Ag diffused into the LNs in a size-dependent manner seems to accumulate only at the proximal ends near the afferent lymphatic vessels, whereas Ag carried by migratory DCs penetrates deep into the medullary zone 128. On the other hand, vaccine Ag transferred from vaccinated muscle cells, KCs, fibroblasts and other tissue cells to skin-resident DCs in the epidermis and dermis is reported to facilitate a durable immune response under limited dosage of vaccine inoculation 32, 33 (**Figure 6A**). Therefore, antigen transfer to DCs is of physiological and clinical significance.

The bivalent (2vHPV, Cervarix), quadrivalent (4vHPV, Gardasil) and nine-valent (9vHPV, Gardasil 9) human papillomavirus (HPV) vaccines are primarily composed of noninfectious virus-like particles (VLP) that display potent protection against cervical infections caused by HPV, condylomas and some HPV-related cancers 212-214, 234, 235. Recently, accumulating evidence indicates that muscle cells at site of injection may act as Ag reservoirs/donors for DCs during i.m. administration to promote the establishment of a sustained anti-viral effector and memory immune defense 236-239. Antigen transfer is also involved in other protein-based vaccines and contributes to an efficient disease prevention and control. Rappazzo et al. 240 reported influenza vaccines based on bacteria-derived outer membrane vesicles (OMVs) and ectodomain of the influenza M2 protein (M2e). Briefly, OMVs were engineered to display M2e by transforming E. coli with a plasmid encoding the transmembrane protein ClyA followed by the Ag of interest, which elicited high IgG titers and protects against lethal doses of the mouse-adapted H1N1 influenza strain PR8 in BALB/c mice, probably due to the OMVs-mediated Ag transfer to APCs *in vivo*.

Patel et al. 241 reported a biocompatible particulate protein delivery system that may exploit the phenomenon of Ag transfer from tumor cells to DCs for improved immunization. Briefly, plasma membrane vesicles (PMVs) are prepared from biological materials (such as cultured cells and isolated tissues) and surface-modified by glycosylphosphatidylinositol (GPI)-anchored TAAs (breast cancer Ag: human epidermal growth factor receptor-2 (HER-2) in this work) and immunostimulatory molecules (such as interleukin (IL)-12 and B7-1), which induced both cellular and humoral immunity against a HER-2-expressing tumor cell challenge along with delayed tumor growth and partial regression of established tumors.

DCs-derived exosomes (Dex) are loaded with costimulatory molecules, functional p-MHCs and other immune cell-interacting elements, and are especially enriched in p-MHC class II complexes, by 10-100-fold that of DCs, which might lead to a more efficient Ag transfer to other DCs and remarkable immunological impacts 242. It should be noted that the immune effects (i.e., stimulation or inhibition) and biological activity of Dex depend on the activation status of donor DCs and the follow-up artificial manipulation of the isolated endosomes. For instance, compared with that from immature DCs, Dex from mature murine DCs are enriched in MHC class II, costimulatory B7.2, intercellular adhesion molecule 1 (ICAM-1) and depleted in milk fat globule-epidermal growth factor-factor VIII (MFG-E8), which are 50- to 100-fold more potent in functional T-cell activation both *in vitro* and *in vivo* 243. And the involvement of exosomes in the induction of host defense and immune evasion has been reviewed by Schorey et al. 244 in detail. Indeed, with advances in molecular and cellular biology, such cell-free multifunctional protein delivery platform might have widespread applications in mediating antigen transfer for a desired immune regulation 245-248.

As mentioned before, antigen transfer might also induce immune tolerance and dampen the protective effect of protein-based vaccines. Hadeiba et al. 249 found that peripheral pDCs engulfed subcutaneously injected exogenous Ag in the absence of TLR signals, and subsequently migrated to the thymus in a CCR9-dependent manner to delete Ag-reactive thymocytes and induce immune tolerance. Specifically, pDCs themselves fail to make physical contacts with CD4^+^ T cells, and are incapable of directly inducing T cell proliferation. Nonetheless, pDCs transport and transfer Ag to thymic APCs to abort the activation and clonal expansion of cognate CD4^+^ T cells, inducing Ag-specific systemic tolerance 208. However, the mechanistic details of such Ag transfer in tolerogenic T cell induction needs further exploration. In addition, given that co-stimulatory membrane molecules and immunostimulatory soluble molecules are low-expressed in most non-APCs, the direct transfer of functional p-MHC class I complexes from Ag-pulsed non-APCs to immature DCs may sometimes induce T-cell tolerance and/or exhaustion due to insufficient costimulatory signals 2, 59, 60, 110.

### Nucleic acid-based vaccines

Compared to protein/peptide-based subunit vaccines that are generally inadequate in immunogenicity, nucleic acid-based vaccines have self-adjuvant effect and can act as pathogen-associated molecular patterns (PAMPs) to stimulate pattern-recognition receptors (PRRs, such as TLR-3/-7/-8/-9) for amplified immune responses 32, 250, which have emerged as promising vaccine platforms in anti-cancer and anti-viral immunotherapy 251, 252. And antigen transfer during the inoculation of nucleic acid-based vaccines is increasingly gaining attention for its potential clinical benefits (**Figure 6B**).

Considering the great discrepancy in lymphatic draining system and cell abundance of different vaccination sites, the administration route and delivery vehicle of the nucleic acid of interest significantly shapes the efficiency and duration of vaccine responses. DNA- and mRNA-based vaccines are commonly delivered via i.d. 253, i.m. 254 and s.c. 255, or through the less adopted i.n. 256, i.v. 257, intra-tumoral injection 258, intra-splenic injection 259, and intranasal administration 260.

After administration, Ag-encoding nucleic acids are directly captured, processed and presented by DCs through MHC class I-biased pathway, or indirectly transferred to DCs from transfected somatic cells in the form of exogenous protein/peptide to induce MHC class II-preferred Ag presentation. In addition, it is reported that pDNA-vaccinated somatic cells may **1)** present associated p-MHC class I complexes on the cell surface to be recognized and even cytolyzed by cognate CD8^+^ T cells 261; or **2)** be phagocytized by DCs for further process and presentation 262. For example, Li et al. 49 found that following vaccination with ovalbumin-encoding pDNA (OVA-pDNA, i.m.), CD103^+^/CD8α^+^ DCs obtain antigenic information from transfected KCs via cross dressing to efficiently activate both naïve and memory CD8^+^ T cells. Similarly, after administration, mRNA vaccines are extensively internalized and expressed by muscle cells and KCs, and the resultant Ag protein/peptide can be transferred to nearby APCs for CD8^+^ T cell activation and immune amplification 32, 33.

### DCs-based vaccines

The adoptive transfer of* ex vivo-*activated DCs has been widely recognized with good biosafety and sufficient efficacy in clinical anti-tumor therapy 263. Briefly, CD34^+^ bone marrow progenitors or CD14^+^ peripheral blood monocytes are isolated and stimulated with granulocyte-macrophage colony stimulating factor (GM-CSF) and IL-4 *in vitro* to generate mo-DCs 264, 265. Then, the resultant mo-DCs are pulsed with **1)** TAAs and/or TSAs 266, 267; **2)** tumor whole cell lysates 268; or **3)** tumor-derived EVs 269, 270, and concurrently stimulated with adjuvants for maturation 271. However, the clinical efficacy of mo-DCs-based vaccine is greatly limited by its inferior ability of CCR7-dependent LNs homing and Ag cross presentation 272. As a result, a large proportion of mo-DCs remained at the site of administration, lost viability and are eliminated by phagocytes 273. In this consideration, some current clinical trials have used enriched cDCs and pDCs that directly collected from the peripheral blood and activated *in vitro* before administration, which might have broader immunotherapeutic applications as these DCs subsets display superior LNs homing and T-cell cross priming 274-276.

**Figure 7:**
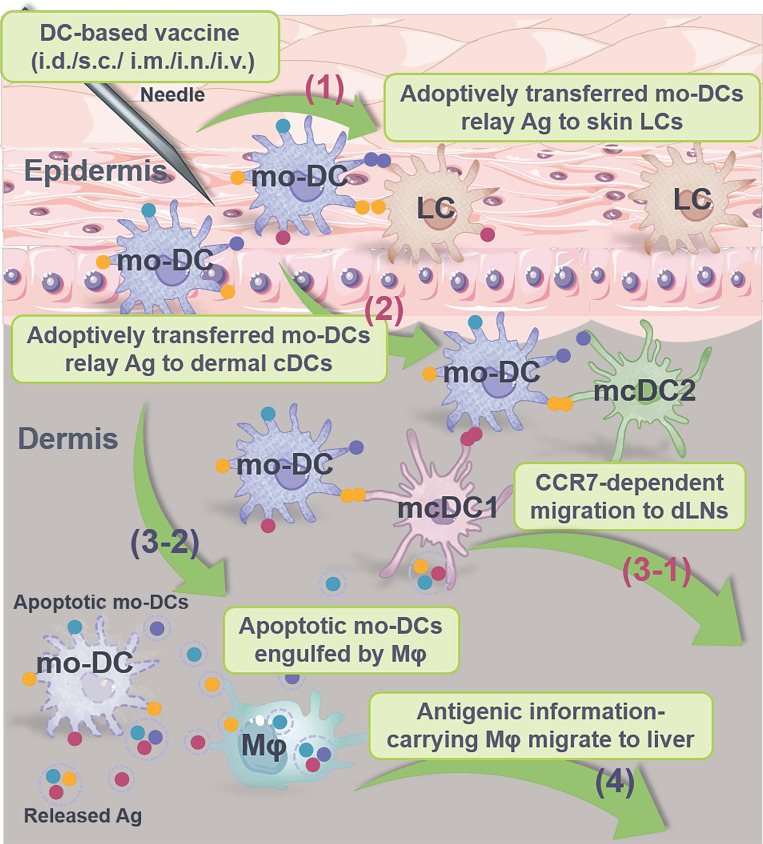
**Antigen transfer in DC-based vaccines.** After local injection, adoptively transferred DCs (as represented by monocyte-derived DCs (mo-DCs)) transfer Ag to Langerhans cells (LCs) in the epidermis *(1)* and migratory conventional DCs (mcDCs) in the dermis *(2)*. Then, part of Ag-loaded mo-DCs, LCs and mcDCs migrate to the dLNs in a CCR7-dependent manner to activate adaptive immune responses *(3-1)*. In addition, mo-DCs that undergo apoptosis *in situ* and their apoptotic bodies are mainly phagocytosed by macrophages *(3-2)* and transported to the liver for immune activation *(4)*.

Nevertheless, multiple clinical phase II and phase III studies have shown that the less mobilized mo-DCs are sufficient in eliciting potent immune responses in cancer therapy 268, 276-278, which may attribute to the antigen transfer from mo-DCs to other APCs both at the site of injection and the dLNs 29, 273, 279 (**Figure 7**). For example, Huang et al. 174 found that even undifferentiated monocytes loaded with Ag protein or peptide induced robust CD8^+^ T cell responses by Ag transfer to endogenous splenic CD8^+^ DCs in a cell-to-cell contact-dependent fashion and through Cx43-mediated intercellular gap junctions. On the other hand, DCs retain at the injection site may transfer Ag to tissue-resident LCs or circulating cDCs through multiple contact- and non-contact- dependent pathways including EVs and trogocytosis to sensitize the immune system for specific activation 72. Meanwhile, dead cells, cell derbies and apoptotic bodies of those DCs can be phagocytized by infiltrating CD163^+^ macrophages as an approach of passive Ag transfer. Consequently, Ag-laden macrophages migrate to the liver for further Ag sharing and T-/B-cell activation 273. In short, intercellular antigen transfer during the administration of DCs-based vaccines may serve as an efficacious strategy to amplify immune response.

## Conclusions and Discussion

In this review, mainly focused on DCs-based antigen receptors, we summarize the recent understanding of antigen transfer and its impact on immune amplification and tolerance. We conclude that antigen transfer plays an important role in coordinating immune responses against the invasion of the “non-self”. Therefore, appropriately manipulating antigen transfer may promote the preventive and/or therapeutic effects of vaccines, which depends heavily on a rational design of the vaccine component and administration route. Meanwhile, undesired antigen transfer may induce tolerance or cause allergy, graft rejection and autoimmune diseases. In these regards, reasonable intervention that blocks or disturbs antigen transfer is needed.

Of note, antigen transfer-associated immune amplification and tolerance can sometimes be interconvertible. In fact, persistent and/or excessively/insufficiently-dosed Ag stimulation may induce a tolerogenic phenotype of DCs that leads to vaccine failure. To avoid the induction of unwanted tolerance to an antigen of interest, **1)** adjuvants that facilitate the recruitment, mobilization, and maturation of DCs can be supplemented; **2)** the route of vaccine delivery that determines the participants of antigen transfer needs further consideration; **3)** the dose and type (i.e., protein-, nucleic acid-, or cells- based vaccines) of vaccine should be carefully selected as different mechanisms of antigen transfer may be involved.

Up to now, the key steps and mediators directing the intercellular antigen transfer remain obscure, and the immunological and pathological consequences of antigen transfer in different biological processes require further exploration. Therefore, more efforts are needed for proper regulation over the mode, site and participant of antigen transfer that might contribute to a more satisfactory immune outcome.

## Figures and Tables

**Table 1:** Antigen transfer with APCs-based receptors

Donor cell	Acceptor cell	Pathway	Ag form	Location	Immunological outcome	Ref.
**APCs to APCs**						
Migratory cDC1	LNs-resident cDC1/2	TNTs, EVs, trogocytosis, gap junctions	p-MHC I/II	LNs	Initiate anti-tumor immune response	1, 48, 55, 134
pDCs	cDC1	EVs	antigen protein/peptide, or p-MHC I	LNs	Cross prime CD8^+^ T cells and induce durable immunity	132, 133
B cells and FDCs, respectively	FDCs and B cells, respectively	EVs	p-MHC II	Follicle	Immunocomplexes deposit on FDCs and cognitive B cells differentiation	183, 184
LCs	Dermal cDCs	EVs, trogocytosis, gap junctions, TNTs	Processed Ag and intact p-MHCs	Skin	Induce immune defense against HSV	152
B cells	mo-DCs	Possibly by EVs, trogocytosis, gap junctions, TNTs	Processed Ag and intact p-MHC II	/	Mo-DCs obtain processed Ag to activate T cells	280
Macrophages	DCs	Gap junctions	Dietary Ag	Intestine	Establish oral tolerance	52, 206
Macrophages	DCs	Gap junctions, EVs	Ingested or processed Ag	Intestine and skin	Resist the infection by Mycobacterium, Salmonella, Listeria and other pathogens	176, 178, 211
Macrophages	B cells	Possibly by gap junctions, TNTs	p-MHCs	Lymphoid follicles	Initiate the early activation of cognate B cells	183
B cells	B220^+^ Macrophages	EVs	Processed Ag fragments or Ag particles	Peritoneum	Macrophages acquire the ability to activate CD4^+^ T cells	187
cDCs	B cells	Possibly by gap junctions, TNTs	Processed Ag fragments, Ag particles and intact p-MHC II	Lymphoid follicles	Activate cognate B cells	180, 185
**Non-APCs to APCs**						
Gene edited 4T1/B16 tumor cells with high expression of MHC I/II	Tumor infiltrating cDC1	Possibly by EVs, trogocytosis, gap junctions, TNTs	p-MHC I/II	Tumor site	Activate tumor specific CD4^+^ T cells	189
Fibrosarcoma tumor cells	cDC2	Possibly by EVs, trogocytosis, gap junctions, TNTs	p-MHC I	Tumor site	Promote antitumor CD8^+^ T cell immunity	190
Melanoma cells and epithelial cells near the colorectal tumor	pDCs	Possibly by EVs, trogocytosis, gap junctions, TNTs	p-MHC I	Tumor site	Compensate the poor cross presentation and phagocytic ability of pDCs	191
Tumor cells and commensal bacteria, respectively	Intestinal commensal bacteria and DCs, respectively	Possibly by EVs, TNTs, trogocytosis	p-MHC I	Tumor site, intestine	Upregulate reactive IFN-γ^+^ T cells and sensitize immune checkpoint blockade efficacy	202-205
UVB irradiated mutate melanocytes	Skin-resident DCs and tumor infiltrating DCs	Possibly by EVs, trogocytosis, gap junctions, TNTs	Possibly p-MHC I	Tumor site, mutated skin	Promote the cure rate of malignant melanoma	281
HCV or HCV infected hepatocytes	pDCs	Contact-dependent gap junctions, TNTs, EVs	HCV RNA	HCV infected liver	Triger TLR 7 activation induced type-I IFN release by pDCs to inhibit HCV infection	192, 193
KCs	Multiple DCs subsets in skin and LNs	Possibly by EVs, trogocytosis, gap junctions, TNTs	Ag-encoding mRNA and protein	Vaccine injection site and draining LNs	Induce an enhanced immune response without immune cell depletion upon repeated inoculation of mRNA vaccine	32
Muscle cells	Mo-DCs	Possibly by trogocytosis, gap junctions, TNTs	mRNA transfected Ag fragments and/or p-MHC I	Vaccine injection site	Elicit potent Ag-specific CD8^+^ T cell immune responses	33
KCs	LCs	TNTs	Ag-encoding mRNA and protein	Vaccine injection site	Promote vaccine effect	161, 162
Symbiotic bacteria and IECs	IECs, macrophages, and DCs	Gap junctions, EVs	Ag fragments	Intestine	Maintain intestinal homeostasis	136, 142, 282
mTECs	Thymus-resident CD8α^+^ DCs	EVs	p-MHC I/II	Thymus	Establish central tolerance	115, 122
Graft cells	DCs in organ recipients	Trogocytosis, EVs	p-MHC I	Transplanted organ	Induce activation and proliferation of allergen-reactive T cells	107, 147, 148
Mast cells	DCs	EVs	Possibly ingested and/or processed Ag fragments, Ag particles, and intact p-MHC II	Near the allergic site	Induce acute inflammatory injury, such as severe vascular leakage, at the allergic sites	144
Epithelial cells	DCs	Possibly by EVs, trogocytosis, gap junctions, TNTs	Possibly ingested and/or processed Ag fragments, Ag particles, and intact p-MHC II	Allergic skin	Cause allergen-associated Th2 immune responses	145
Platelets	DCs, Macrophages	EVs	Mitochondria DNA and multiple autoantigens	Kidney	Aggravate systemic lupus erythematosus	146

**Table 2:** Antigen transfer and its immunological effects by different types of vaccine

Vaccine type	Vaccine component	Administration route	Major Ag donor cells	Major Ag receptor cells	Immunological outcome	Ref.
**Protein-based vaccine**						
Protein	Multivalent HPV protein Ag and adjuvant AS04	i.m.	Muscle cells and skin-resident DCs, respectively	Skin-resident DCs and LNs-resident DCs, respectively	Prevent HPV induced infections and cancers	212-214
Protein	5-20 recombinant/fusion tumor neoantigens	s.c.	KCs and skin-resident DCs, respectively	Skin-resident DCs and LNs-resident DCs, respectively	71.4 % of cancer patients are under control with specific CTL response elicited	236
Protein	TAAs (HER-2) and immunostimulatory molecules modified plasma membrane vesicles (PMVs)	s.c.	Breast cancer cells	DCs in subcutaneous compartment and LNs	Induce both cellular and humoral immunity against HER-2-expressing tumor cells	241
Protein	M2e-displaying outer membrane vesicles (OMVs)	s.c.	Escherichia coli	Skin somatic cells and DCs	Initiate specific humoral immunity against influenza A (H1N1)	240
Protein	Oligodendrocyte-derived EVs containing multiple myelin Ags	i.v.	Oligodendrocyte, monocyte, cDCs	mo-DCs	Induce immunosuppressive monocytes and apoptosis of autoreactive CD4^+^ T cells in several autoimmune encephalomyelitis models	283
Protein	OVA	s.c.	Skin somatic cells and CCR9^+^ pDCs, respectively	CCR9^+^ pDCs and thymus cDCs, respectively	Induce pDCs-mediated thymic central tolerance	249
**Nucleic acid-based vaccine**						
pDNA	OVA pDNA	i.m.	KCs	CD103^+^/CD8α^+^ DCs	Activate OVA-specific CD8^+^ T cells	49
pDNA	Bacillus anthracis protective antigen domain 4 (PA-D4) pDNA	i.d. by electroporation	KCs	Skin-resident DCs	Induce potent Anthrax-associated humoral immune response	284
pDNA	OVA pDNA and GM-CSF -loaded mesoporous silica microrods (MSRs)	s.c.	KCs and migratory DCs, respectively	Skin-resident DCs and LNs-resident DCs, respectively	Elicit OVA-specific CTL response, Th1 humoral response and CD8^+^ effector and memory T cell responses	285
mRNA	Influenza A mRNA delivered by Lipofectamine 2000	i.m.	Muscle cells	mo-DCs	Cross prime CD8^+^ T cells *in vivo*	33
mRNA	Protamine mRNA	i.d.	KCs and migratory DCs, respectively	Migratory DCs and LNs-resident DCs, respectively	Induce functional Ags in the dLNs and massive activation of T cells	32
**DCs-based vaccine**						
mo-DCs	Mo-DCs loaded with both keyhole limpet hemocyanin (KLH) and TAA	i.d., i.n.	mo-DCs	CD163^+^ macrophages and LNs-resident DCs	Induce Ag-specific immune response in patients with melanoma	273
mo-DCs	*In vivo* activated mo-DCs	s.c.	mo-DCs	LNs-resident CD8α^+^ DCs	Activate B16-OVA specific CD8^+^ T cell immune response	279
mo-DCs	Tumor whole cell lysate-pulsed mo-DCs	i.d.	mo-DCs	Possibly DCs and macrophages in the dLNs and vaccine injection site	Nearly half of the patients generate specific immune responses against glioblastoma, with survival time prolonged	268, 277
mo-DCs	Tumor whole cell lysate-pulsed mo-DCs	s.c.	mo-DCs	Possibly DCs and macrophages in LNs and vaccine injection site	Induce renal cell cancer-specific Th1 immune response	286
cDC2 and pDCs	Three TAAs/mRNA-pulsed cDC2 and pDCs	i.d.	cDC2 and pDCs	LNs-resident DCs	Increase metastatic castration-resistant prostate cancer (mCRPC) reactive IFN-γ^+^ CTLs	276
cDC2	TAAs (gp100 and tyrosinase) -pulsed cDC2	i.d.	cDC2	LNs-resident DCs	Prolong progression free survival in some melanoma patients	274
pDCs	TAAs (gp100 and tyrosinase) -pulsed pDCs	intra-LN	pDCs	LNs-resident DCs	Prolong the survival of melanoma patients with 1-2 years	275
pDCs	Peripheral Ag (OVA) -loaded pDCs	i.v.	CCR9^+^ pDCs	Thymus-resident cDCs	Induce central tolerance	249

## Abbreviations

Ag: antigen
p-MHCs: peptide-MHC complexes
SLOs: secondary lymphoid organs
TNTs: tunnel nanotubes
EVs: extracellular vesicles
DCs: dendritic cells
APCs: antigen-presenting cells
Tregs: regulatory T cells
cDCs: conventional DCs
LCs: Langerhans cells
pDCs: plasmacytoid DCs
mo-DCs: monocyte-derived DCs
LNs: lymph nodes
PPs: Peyer's patches
KCs: keratinocytes
HSV: herpes simplex virus
EBV: Epstein-Barr virus
CTL: cytotoxic T lymphocyte
TAAs: tumor-associated antigens
TSAs: tumor-specific antigens
HIV: human immunodeficiency virus
HLA-G: human leukocyte antigen-G
MDSCs: myeloid-derived suppressive cells
FDCs: follicular DCs
Cx43: Connexin 43
dLNs: draining lymph nodes
F-actin: filamentous actin
CCR7: chemokine receptor 7
MVEs: multivesicular endosomes
EVIR: extracellular vesicles-internalizing receptors
mTECs: medullary thymic epithelial cells
AIRE: autoimmune regulator
SCS: subcapsular sinus
SFB: Segmented filamentous bacteria
IECs: intestinal epithelial cells
Th17: T helper type 17
Mφ: macrophages
MCs: Mast cells
MCGs: mast cell granules
VPS4: vacuolar protein sorting 4
AD: atopic dermatitis
Th2: T helper type 2
HDM: house dust mite
DAMPs: danger-associated molecular patterns
TLRs: Toll-like receptors
SLE: systemic lupus erythematosus
mtDNA: mitochondrial DNA
PD-L1: programmed death-ligand 1
CLEC9A: C-type lectin-like receptor 9A
EVIR: extracellular vesicle-internalizing receptor
NK: natural killer
CEA: carcinoembryonic antigen
Th1: T helper type 1
NIPCs: natural interferon producing cells
IFN: type I Interferon
Lm: Listeria monocytogenes
MPs: microparticles
BCRs: B cell antigen receptors
LOX-1: Lectin-like oxidized low-density lipoprotein receptor-1
mcDCs: migratory conventional DCs
rcDCs: resident conventional DCs
IRF8: interferon regulatory factor 8
IRF4: interferon regulatory factor 4
HCV: hepatitis C virus
mRNA: messenger RNA
E. coli: Escherichia coli
HepA/B: hepatitis A/B
SARS-CoV-2: severe acute respiratory syndrome coronavirus 2
pDNA: plasmid DNA
HPV: human papillomavirus
VLP: virus-like particles
OMVs: outer membrane vesicles
M2e: influenza M2 protein
PMVs: plasma membrane vesicles
GPI: glycosylphosphatidylinositol
HER-2: human epidermal growth factor receptor-2
IL: interleukin
Dex: DCs-derived exosomes
ICAM-1: intercellular adhesion molecule 1
PAMPs: pathogen-associated molecular patterns
PRRs: pattern-recognition receptors
GM-CSF: granulocyte-macrophage colony stimulating factor
